# A Combinational Optimization Method for Efficient Production of Indigo by the Recombinant *Escherichia coli* with Expression of Monooxygenase and Malate Dehydrogenase

**DOI:** 10.3390/foods12030502

**Published:** 2023-01-21

**Authors:** Zijing Pan, Dejiang Tao, Mingjing Ren, Lei Cheng

**Affiliations:** Beijing Engineering and Technology Research Center of Food Additives, Beijing Advanced Innovation Center for Food Nutrition and Human Health, Beijing Technology and Business University (BTBU), Beijing 100048, China

**Keywords:** indigo pigment, heterologous expression vector, promoter optimization, biosynthesis

## Abstract

Indigo pigment is a widely used pigment, and the use of biosynthesis to ferment indigo has become a hot research topic. Based on previous research, the indigo could be biosynthesized via the styrene oxygenation pathway, which is regulated by intracellular redox-cofactor rebalancing. In this work, the malate dehydrogenase (*mdh*) gene was selected as an NADH regeneration element to improve the intracellular cofactor regeneration level, and it was co-expressed with the styrene monooxygenase (*styAB*) gene by pET-28a(+) vector in *E. coli* for enhancing indigo production. The *P_T7_* and *P_cat_* promoter was constructed to change the *styAB* gene and *mdh* gene from inducible expression to constitutive expression, since the expressing vector pET-28a(+) needs to be induced by IPTG. After different strategies of genetic manipulations, the *styAB* gene and *mdh* gene were successfully constitutively co-expressed by different promoters in *E. coli*, which obviously enhanced the monooxygenase activity and indigo production, as expected. The maximum yield of indigo in recombinant strains was up to 787.25 mg/L after 24 h of fermentation using 2.0 g/L tryptophan as substrate, which was nearly the highest indigo-producing ability using tryptophan as substrate in recent studies. In summary, this work provided a theoretical basis for the subsequent study of indigo biosynthesis and probably revealed a new insight into the construction of indigo biosynthesis cell factory for application.

## 1. Introduction

Indigo is a blue and odorless powder, which belongs to the natural indole reductive dye [1]. Technologies for indigo production have been achieved by extraction from plants and chemical synthesis for many years [2,3,4]. However, chemical methods for indigo synthesis would result in major environmental pollution because of the use of toxic chemicals, and indigo production from plants also shows obvious disadvantages in cost and yield [5,6,7]. Therefore, indigo preparation by microbial methods has been widely considered by researchers and may overcome the limitations of plant extraction and chemical synthesis methods.

Previous studies showed that most of the microorganisms that exhibit the ability to synthesize indigo pigment are aromatic hydrocarbon–degrading strains, including *Pseudomonas* sp. [8,9,10,11,12], *Rhodococcus* sp. [13], *Comamonas* sp. [14], *Acinetobactor* sp. [15] and *Sphingomonas* sp. [16], among which *Pseudomonas* is the most reported genus. These strains can degrade aromatic hydrocarbons containing a variety of dioxygenase and monooxyase [17]. These oxygenase-catalyzed oxygenation (hydroxylation) reactions need to be reduced by different types of cofactors, such as NADP^+^, FAD, ascorbic acid, cytochrome C, Cu^2+^, and F^2+^ [18]. Although the wild strain could produce indigo, it could not meet the needs of modern industrial synthesis due to its low yield [19]. Therefore, the heterologous expression of key enzymes by recombinant strains has become a research focus, which solves the problems of optimizing cultivation conditions and the long fermentation period of the wild strain, and thus significantly improves the output of indigo [20,21].

In our previous study, we isolated an indigo-producing strain of *Pseudomonas putida* B3. Moreover, we verified that the styrene monooxygenase (IMO) encoded by the styrene monooxygenase gene (*styAB*) was leading the indigo biosynthesis pathway using indole as the substrate, and the whole biosynthesis was regulated by the rebalancing of NADH/NAD^+^ [22,23]. However, the high concentration of indole had certain toxic and side effects on biological cells, and the growth of bacteria was inhibited, resulting in low production of indigo pigment [24,25]. Thus, studies were reported that tryptophan could be used as substrate for indigo production when the *styAB* gene was heterologously expressed in *Escherichia coli*, due to the tryptophan-related gene (*tnaA*) in *E. coli*, which can catalyze the conversion of tryptophan into indole [26]. The pathway of indigo conversion from tryptophan in *E. coli* is shown in Figure S1. Firstly, the tryptophan is degraded by tryptophanase to form indole. Then, cis-indole-2,3-dihydrodiol is generated from indole through dehydration and oxidation under the regulation of monooxygenase. Finally, 3-hydroxyindoles are dimerized into indigo.

In this work, we heterologously expressed the *styAB* gene in *E. coli* using the pET vector system. For improving the efficiency of rebalancing NADH/NAD^+^ and increasing the production of indigo, different strength and regulatory features of promoters were used for co-expression optimization of the *styAB* gene and the malate dehydrogenase gene (*mdh*) by changing the inducible expression to constitutive expression, according to the characteristics of the redox reaction in the synthesis pathway of indigo. The genetic manipulation strategy probably revealed a new insight into the construction of indigo biosynthesis cell factory for application.

## 2. Materials and Methods

### 2.1. Enzymes and Chemicals

Indigo pigment standard products, tryptophan, N, N-dimethylformamide, indole and kanamycin were purchased from Sigma–Aldrich (St. Louis, MO, USA); PrimeSTAR Max Premix (2×) DNA polymerase, T4 DNA ligase, nucleic acid restriction quickCut enzyme *Hind*III, *BamH*I, *Mlu*I, *Xba*I, *Sfo*I, *Mlu*I and molecular quality standard DNA Marker, 10,000 DL DNA Marker, were purchased from Takara Bio (Dalian, China). Other reagents are domestic market, pure analysis.

### 2.2. Strains, Plasmids and Culture Conditions

The plasmids and strains used in this research are listed in Table 1. *Pseudomonas putida* B3 was cultured at 30 ℃, and *E. coli* strains were cultured at 37 °C in Luria–Bertani (LB) medium with vigorous shaking. The antibiotic kanamycin was added at the concentration of 50 μg/mL when needed.

### 2.3. DNA Manipulation Techniques

The DNA manipulation procedure was performed as described by Green and Sambrook [27]. Genomic DNA was isolated using TIANamp Bacteria DNA kit (Tiangen Biotech, Beijing, China) according to the manufacturer’s instructions. Plasmids were prepared using the high-purity plasmid small quantity preparation kit (Tiangen Biotech, Beijing, China), following the manufacturer’s instructions. DNA fragments encoding *styAB* gene and *mdh* gene were amplified by PCR using PrimeSTAR Max Premix (2×) DNA polymerase (Takara Bio, Dalian, China) according to the manufacturer’s protocol, with the following cycle profile: 95 °C for 5 min, 40 cycles of 94 °C for 30 s, 53 °C for 1 min, 72 °C for 5 s, and a final extension at 72 °C. Primers used are shown in Table S1.

### 2.4. Shaking Flask Fermentation of Indigo

Fresh cultures of *E. coli* were inoculated into the fermentation medium at a ratio of 1.2%. The fermentation media was composed of disodium 18.0 g/L hydrogen phosphate, 3.5 g/L yeast extract, 3.2 g/L KH_2_PO_4_, 1.2 g/L NH_4_Cl, 0.7 g/L NaCl, and 0.2 g/L MgSO_4_, containing 2.0 g/L tryptophan as substrate. Fermentation was carried out in a fermenter with the parameters set at 200 r/min and the temperature at 37 °C. Samples were collected at 4, 8, 12, 24, 36 and 48 h for determination of indigo concentration. All fermentation data were representative of three independent experiments and performed in triplicate.

### 2.5. Gene Expression Optimization of styAB and mdh by Using Different Promoters

The *styAB* and pET-28a(+) fragments were subjected to double digestion by TaKaRa Quickcut *BamHI* and *HindIII* restriction endonucleases. The conditions of enzyme digestion reaction were at 37.0 °C for 20 min. The extracted gene fragments and vector fragments were linked by TaKaRa DNA Ligation Kit Ver 2.1 (Takara Bio, Dalian, China). After incubation at 16 °C for 30 min, the product of ligation was pET-28a(+)-*styAB*, which was transferred into BL21(DE3)-receptive cells and then named E211. *P_cat_* promoter, *P_T7_* promoter and pET-28a(+)-*styAB* were subjected to double digestion by TaKaRa Quickcut *MluI* and *XbaI* restriction endonucleases. The conditions of enzyme digestion reaction were at 37.0 °C for 15 min. After that, the digested *P_T7_* promoter fragments were connected with digested pET-28a(+)-styAB fragments, and the digested *P_cat_* promoter fragments were connected with digested pET-28a(+)-styAB fragments, respectively. After that, constitutive expression vectors pETP_T7_-styAB and pETP_cat_-styAB were constructed successfully and transferred into BL21(DE3)-receptive cells, then named E212 and E213.

Using *mdh* and *P_T7_* promoter as templates, primers F-*mdh*-up-*P_T7_* and R-*mdh*-up-*P_T7_*, and F-*mdh*-down-*P_T7_* and R-*mdh*-down-*P_T7_*, were used to amplify Δ*mdh* and Δ*P_T7_* gene combinations, respectively. DNA fragments encoding Δ*mdh* and Δ*P_T7_* were amplified by polymerase chain reaction (PCR) using the primers shown in Table S1 with PrimeSTAR Max Premix (2×) DNA polymerase, according to the manufacturer’s recommendations with the following cycle profile: 95 °C for 5 min, 30 cycles of 94 °C for 1 min, 56 °C or 59 °C for 30 s, 72 °C for 30 s or 6 s, and a final extension at 72 °C for 10 min. The concentrations of Δ*mdh* and Δ*P_T7_* were determined, and the PCR products were mixed into the reaction system to obtain the combined fragments of *P_T7_*-*mdh* with a molar ratio of 1:1 SOE-PCR system: PrimeSTAR Max Premix (2×) added 12.5 µL, Δ*mdh* added 0.8 µL, Δ*P_T7_* added 1 µL, and ddH_2_O added 8.7 µL, according to the manufacturer’s recommendations with the following cycle profile: 95 °C for 5 min, 6 cycles of 94 °C for 1 min, 59 °C for 30 s, and 72 °C for 10 s. After 6 cycles, the reacted system was placed on ice to cool for 5 min, and primers F-*mdh*-up-*P_T7_* and R-*mdh*-down-*P_T7_* were added with 1 µL each. Then the PCR protocol was performed again, and the reaction procedure was as follows: 95 °C for 5 min, 24 cycles of 94 °C for 1 min, 58.5 °C for 30 s, 72 °C for 10 s, and a final extension at 72 °C for 2 min. In the same way, *mdh* gene and promoter *P_cat_* were used as templates, and primers F-*mdh*-up-*P_cat_*, R-*mdh*-up-*P_cat_*, F-*mdh*-down-*P_cat_* and R-*mdh*-down-*P_cat_* were used to construct the original *P_cat_*-*mdh* combination fragments.

The one-step cloning method was used to construct recombinant vectors pETP_T7_-styAB-*P_T7_*-*mdh*, pETP_T7_-styAB-*P_cat_*-*mdh*, pETP_cat_-styAB-*P_T7_*-*mdh* and pETP_cat_-styAB-*P_cat_*-*mdh*. ClonExpress II One Step Cloning Kit C112 from Vazyme Biotech (Nanjing, China) was used for connection according to the manufacturer’s recommendations. We used known DNA sequences of pETP_T7_-styAB vector, upstream primers F-pETP_T7_-MluI-*P_T7_*-*mdh* and downstream R-pETP_T7_-SfoI-*P_T7_*-*mdh* primers, which were designed to construct linearized vector fragments with homologous arms. Then the linearized pETP_T7_-styAB vector fragments were mixed with *P_T7_*-*mdh* combination fragments and the reaction system was calculated. The connection reaction was performed with the following cycle protocol: 95 °C for 5 min, 30 cycles of 94 °C for 1 min, 59 °C for 30 s, 72 °C for 6 s, and a final extension at 72 °C for 1 min. After the connection reaction, the recombinant vectors pETP_T7_-styAB-*P_T7_*-*mdh* were obtained and transferred into BL21(DE3)-receptive cells, namely, E214. Following the same process, the recombinant strains E215, E216 and E217 would be acquired also.

### 2.6. RNA Preparation and Quantitative Real-Time Reverse Transcription PCR (qRT-PCR) Analysis

Total RNA was isolated from cells grown in fermentation medium using the Ultrapure Total RNA rapid extraction kit (Tiangen Biotech, Beijing, China). After extracting total RNA, the FastQuant cDNA first strand synthesis kit (Tiangen Biotech, Beijing, China) was used for reverse transcription, following the manufacturer’s instructions. qRT-PCR was carried out using SuperReal PreMix Plus (Tiangen Biotech, Beijing, China) with the following conditions: 95 °C for 5 min, followed by 45 cycles of 95 °C for 10 s, 60 °C for 15 s, and 72 °C for 20 s. Primers used are shown in Table S1, and relative expression levels were calculated by the 2^−ΔΔCt^ method using the 16 s rDNA as the internal control gene. All reactions were performed in triplicate.

### 2.7. Measurement of Growth Curve

The constructed strains were inoculated into LB medium and cultured at 37.0 °C to prepare seed liquid. BioLector Ⅰ micro-bioreactor (M2P, Baesweiler, Germany) was used for online continuous biomass detection, and the growth curve of strains in seed liquid was obtained.

Due to the production of indigo pigment in the fermentation process, plate counting was used to obtain the total number of cells, and the growth curves of all engineering bacteria in the fermentation medium were determined as follows: after the seed liquid was inoculated in the fermentation medium, appropriate bacterial liquid was diluted every 4.0 h and then coated in the solid fermentation medium, with 3 parallel coatings at each time, and cultured at 37 °C for 24 h. Single colony was counted on a plate with sampling time as abscissa and colony count as ordinate to draw the growth curve.

### 2.8. Enzyme Assays

Indole monooxygenase (IMO) activity was measured via indole consumption as described previously [22]. Cells were harvested by centrifugation at 10,000 r/min for 10 min at 4 °C, washed twice with 50 mM potassium phosphate buffer (pH 7.0), and resuspended in the same buffer containing 50 mM indole. Then, 20 mL of cell suspension was transferred into a 50 mL tube and incubated in a shaking water bath at 30 °C, and the indole depletion was monitored every 5 min. Samples were analyzed after filtration; 1 µmol of indole depletion in 1 min was defined as 1 unit (U) of IMO activity.

The malate dehydrogenase (MDH) activity was analyzed by investigating the absorbance at 340 nm, corresponding to the reduction of NAD^+^ using a UV-visible spectrophotometer (SHIMADZU UV-3600, Kyoto, Japan). The cells were harvested at 13,000 r/min for 10 min at 4 °C after fermentation. Then the cells were washed with 67 mM phosphate buffer (pH 7.4) twice. Thereafter, cells were resuspended to a final density of 30 (OD_600nm_) with the same buffer. The supernatant was obtained by centrifuging at 13,000 r/min for 10 min at 4 °C to determine the MDH activity.

The NADH/NAD^+^ ration was measured to investigate the ability of redox-cofactor rebalancing. The in vivo concentrations of NADH and NAD^+^ were examined using a regent kit (Solarbio Tech, Beijing, China) and following the manufacturer’s instructions.

### 2.9. Measurement of Indigo and Tryptophan

All the bacterial liquid was collected into the centrifuge tube, centrifuged at 9000 r/min for 25.0 min, and the supernatant was discarded. The precipitation obtained was dissolved with an appropriate amount of N, N-dimethylformamide and treated with ultrasound for 22.0 min and filtered. Indigo was then quantified by HPLC (Agilent 1290, Santa Clara, CA, USA) equipped with an Agilent Eclipse Plus C18 RRHD column (1.8 µm, 2.1 × 5.0 mm) and DAD detector. The mobile phase was water/methanol (10:90, *v*/*v*) and the operating conditions were as follows: detection at 610 nm, and flow rate of 0.2 mL/min. All samples were analyzed in triplicate.

For tryptophan determination, the fermentation liquid was centrifuged at 9000 r/min for 10 min. Then the supernatant was collected and filtrated for determination by HPLC (Agilent 1290, Santa Clara, CA, USA) equipped with an Agilent Eclipse Plus C18 RRHD column (1.8 µm, 2.1 × 5.0 mm) and DAD detector. The mobile phase was 0.03% KH_2_PO_4_ solution/methanol (90:10, *v*/*v*), and the operating conditions were as follows: detection at 278 nm, and flow rate of 0.4 mL/min. All samples were analyzed in triplicate.

## 3. Results

### 3.1. Introduction and Promoter Optimization of Indigo Biosynthetic Pathway Genes into E. coli

The strain designated *E. coli* E211 was generated harboring the vector pET-28a(+)-styAB with indigo biosynthetic pathway gene *styAB*. Then we designed primers with *P_T7_* promoter, to transform the inducible expression into constitutive expression on pET-28a(+). Hereby, recombinant strain *E. coli* E212 harboring the vector pETP_T7_-styAB by inserting *P_T7_* promoter and *styAB* gene was obtained. Likewise, the recombinant strain *E. coli* E213 harboring the vector pETP_cat_-styAB was also constructed.

**Figure 1:**
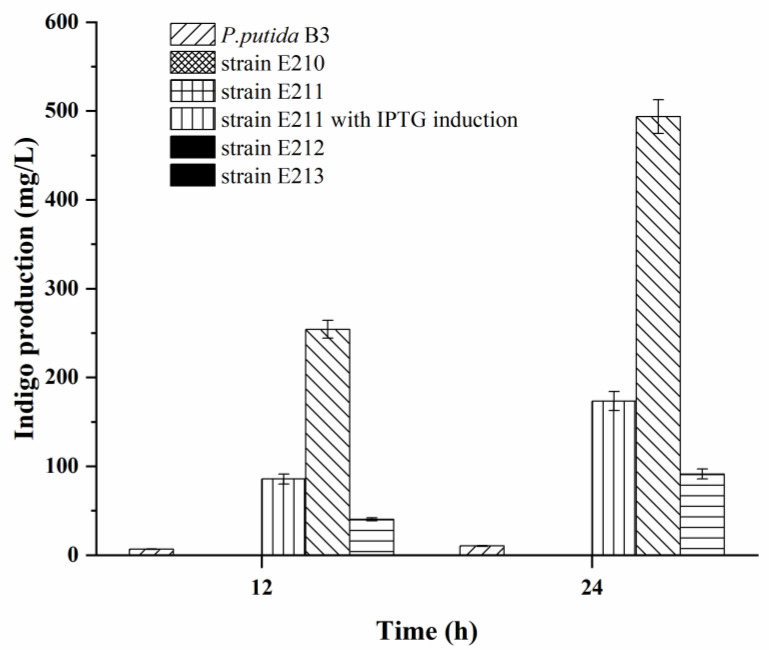
Indigo production from tryptophan at the 2.0 g/L concentration in fermentation of *Psedumononas putida* B3, E211, E212 and E213 after 12 h and 24 h.

In addition, all the recombinant strains were cultured in the fermentation with 2 g/L tryptophan as substrate. Strain E211 was cultured with and without IPTG. Meanwhile, the recombinant strains E212 and E213 were also cultured without any induction. Then the content of indigo in fermentation broth was determined. As can be seen from Figure 1, strain E210 itself did not have the ability to synthesize indigo pigment, and the *styAB* gene was expressed particularly in the strains E211, E212 and 213 to produce indigo pigment. It can be concluded that the *styAB* gene has been successfully introduced into *E. coli* recombinant strains and expressed. Accordingly, the indigo production of the strain E211 after 24 h fermentation with IPTG induction, which was constructed with inducible vector, was 91.33 mg/L and significantly lower than the strains E212 and E213. In this experiment, the constructed plasmid promoter was optimized, and strains E212 and E213 were constructed to change the expression of *styAB* from inducible expression to constitutive expression in the strains. Herewith, without adding any inducer, 493.67 mg/L of indigo was obtained in strain E212 after 24 h of fermentation, which was approximately 48-fold that in the wild strain *Pseudomonas putida* B3. Also, the indigo production of strain E213 after 24 h fermentation was 173.39 mg/L, which was increased by 19 times. It demonstrated that the difference in indigo yield was due to the different promoters used in strains E211, E212 and E213, and the *P_T7_* promoter was able to express *styAB* more efficiently in this research, resulting in more indigo production in the strain E212, which preliminarily achieved the purpose of increasing the indigo yield.

### 3.2. Regulation of styAB Expression by Redox-Cofactor Rebalancing with Promoter Combinational Optimization

For high indigo production through appropriate expression of the *styAB* gene and regulating redox-cofactor rebalancing ability by the *mdh* gene, the strength and regulatory features of promoters should be optimized. The “strong” *P_T7_* promoter and the “weak” *P_cat_* promoter were both first used for co-expression optimization of the *styAB* gene and the *mdh* gene, respectively. Hereby, we respectively co-expressed *P_T7_* promoter ligated with *styAB* gene upstream and *P_T7_* promoter ligated with *mdh* gene upstream in strain E214; *P_cat_* promoter ligated with *styAB* gene upstream and *P_T7_* promoter ligated with *mdh* gene upstream in strain E215; *P_T7_* promoter ligated with *styAB* gene upstream and *P_cat_* promoter ligated with *mdh* gene upstream in strain E216; and *P_cat_* promoter ligated with *styAB* gene upstream and *P_cat_* promoter ligated with *mdh* gene upstream in strain E217.

**Figure 2:**
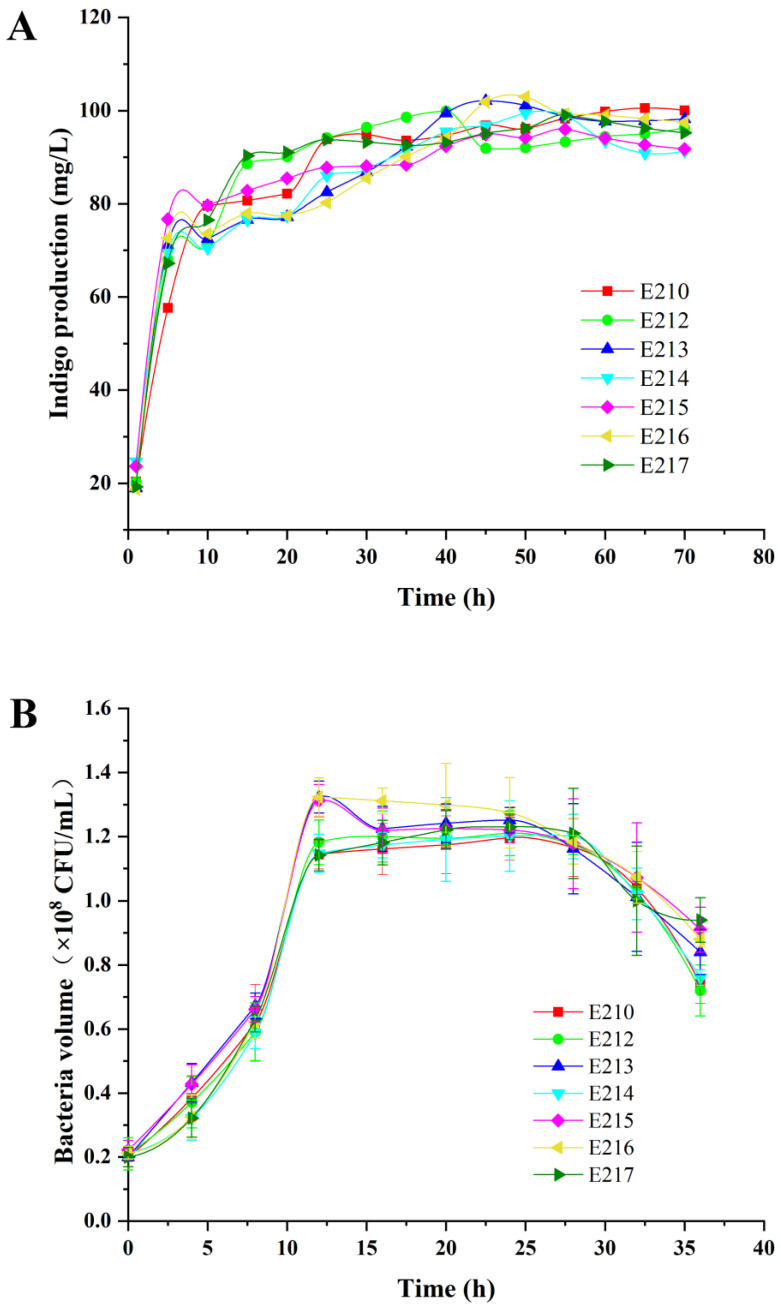
The growth curves of recombinant strains E210, E212, E213, E214, E215, E216 and E217 after different times of culture in LB medium (**A**) and fermentation medium (**B**).

To investigate the expression levels of the *styAB* gene and *mdh* gene in the recombinant strains, the cell growth was monitored first, as shown in Figure 2. After co-expression of the *styAB* gene and *mdh* gene, each recombinant strain shared a highly similar growth tendency under the same culture conditions, indicating that co-expression of the *styAB* gene and *mdh* gene with different promoter combinations had no significant effect on the growth, whether cultured in LB medium (Figure 2A) or in fermentation medium (Figure 2B).

**Figure 3:**
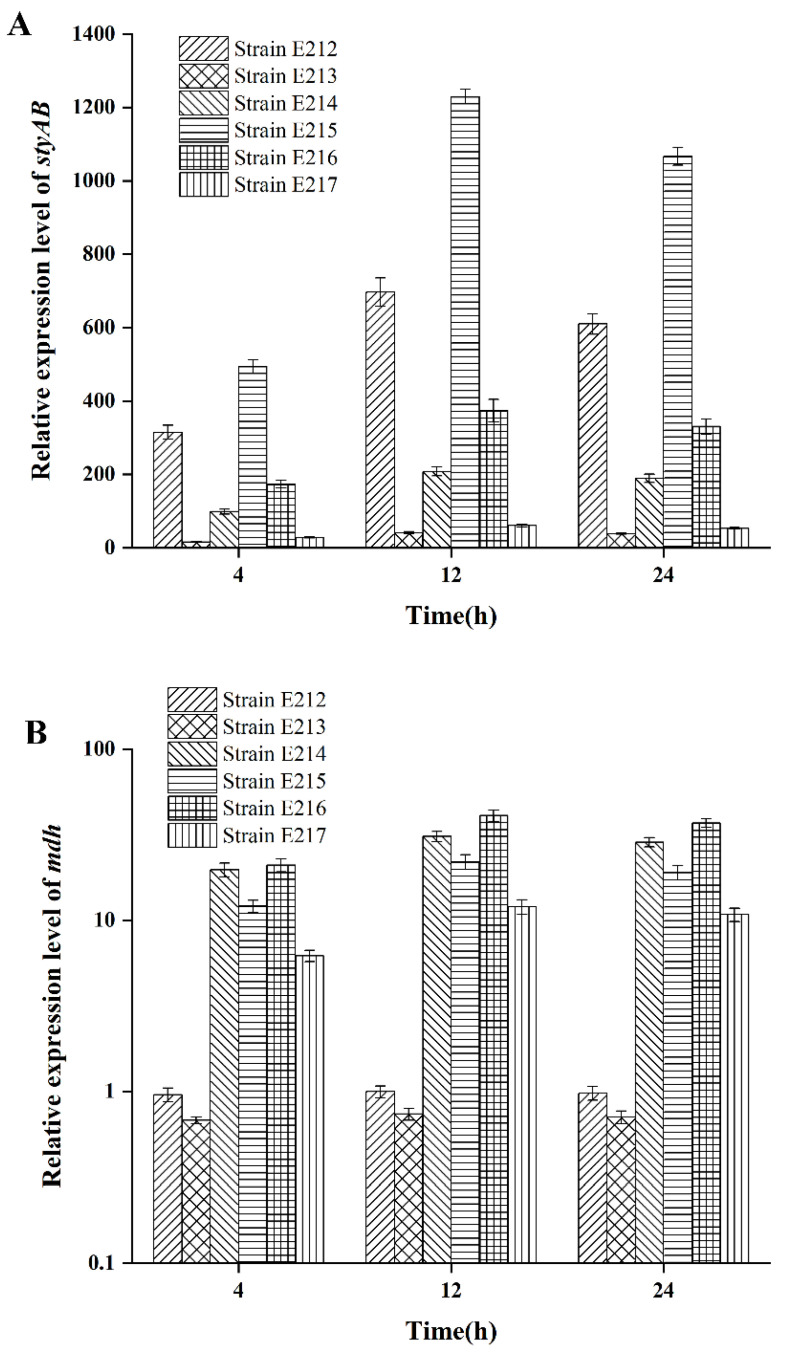
Transcriptional level assay of the genes *styAB* and *mdh* in recombinant strains cultured under fermentation conditions with 2.0 g/L of tryptophan as substrate after 4 h, 12 h and 24 h. (**A**) Expression level of *styAB* gene. (**B**) Expression level of *mdh* gene.

Strains E212, E213, E214, E215, E216 and E217 were individually cultivated in fermentation medium with 2.0 g/L tryptophan as substrate for 4 h, 12h and 24 h, and the relative expression levels of the *styAB* gene and *mdh* gene were measured using the 16s rDNA as the internal control gene. As shown in Figure 3, the relative expression levels of *styAB* in strain E215 were the highest. And the expression level of the *mdh* gene in strains E214, E215, E216 and E217 was higher than that in strains E212 and E213, indicating that the *mdh* gene had been successfully overexpressed in strains E214, E215, E216 and E217. It was clear that the expression of the *styAB* gene was regulated by the *mdh* gene and the rebalancing of NADH/NAD^+^ [22,23]. However, a higher expression level of the *mdh* gene did not necessarily lead to the higher expression level of the *styAB* gene. It was found that the transcriptional level of the *styAB* gene in strains E214 and E216 was reduced compared with that in strain E215, notwithstanding that the *mdh* gene ligated the “strong” promoter *P_T7_* and these two recombinant strains exhibited the ratio of NADH/NAD^+^. This result was probably attributed to the increase of cofactors after the “strong” promoter expressed the *mdh* gene. It is known that the malate dehydrogenase (MDH) regulated by the *mdh* gene mainly produces reducing coenzyme factor NADH. NADH is mainly involved in material and energy metabolism and acts as a carrier of biohydrogen and electron donor to provide reducing power for redox reactions in vivo. As the indigo biosynthesis is not the only pathway that is regulated by NADH in vivo, we presumed that the overexpression of the *mdh* gene would increase the ratio of NADH/NAD^+^ (shown in Figure 3) and then accelerate other intracellular reactions. Due to the enhancement of other metabolic activities, the expression of the *styAB* gene was instead weakened.

### 3.3. Activities of Indigo Biosynthesis Enzymes and Ratio of NADH/NAD^+^

**Figure 4:**
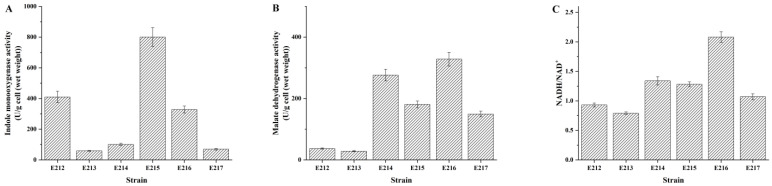
Optimization of the *styAB* gene and *mdh* gene by using different promoters, and effects of different levels of IMO activity (**A**), MHD activity (**B**), and NADH/NAD^+^ (**C**) ratio in vivo by engineered *E. coli* under the fermentation conditions with 2.0 g/L tryptophan as substrate.

The activities of indole monooxygenase (IMO) and malate dehydrogenase (MDH) in the recombinant strains under the fermentation conditions were also assayed. As shown in Figure 4B,C, all recombinant strains with *mdh* gene overexpression exhibited relatively high MDH activities and NADH/NAD^+^ ratio. Combining the analysis results of gene expressions, we found that the “strong” promoter *P_T7_* could exhibit higher enzyme activity of MDH by higher gene expression level, leading to the increase in the ratio of NADH/NAD^+^, as expected. However, it was notable that strain E215 expressed the highest activity of indole monooxygenase (IMO) after 12 h of fermentation, even though the MDH exhibited the highest activity in strain E216. The results of enzymatic activities analysis were quite similar to the results of expression level of *styAB* gene and *mdh* gene in the recombinant strains. We verified that the high ability of regenerating NADH from NAD^+^ brings positive effects on IMO activity, within limits. However, the higher enzyme activity of MDH and the ratio of NADH/NAD^+^ did not always induce the higher activity of IMO. When in vivo MDH activity and NADH/NAD^+^ ratio increased significantly in strains E214 and E216, all metabolic pathways that depend on the redox power were enhanced. It was speculated that the increase of some other enzyme activities, which was prior to the IMO activity, could promote the enhancement of related biological pathways, and may inhibit the expression of the *styAB* gene or reduce the enzyme activity of IMO.

### 3.4. Indigo Production in Recombinant Strains

**Figure 5:**
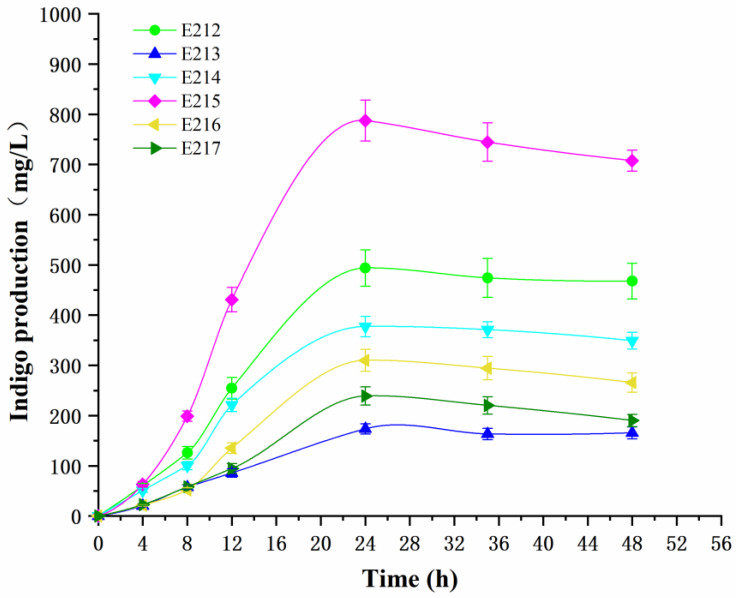
Indigo production of strains E212, E213, E214, E215, E216 and E217 under fermentation conditions with 2.0 g/L of tryptophan as substrate at different times.

In this research, to investigate the effect of co-expression optimization of the *styAB* gene and the *mdh* gene, the indigo production of the recombinant strains cultured under fermentation conditions containing 2.0 g/L tryptophan was determined at 37 °C for 4, 8, 12, 24, 36 and 48 h. The results (Figure 5) showed that indigo production accumulated rapidly as cells proliferated at the beginning in all recombinant strains, and then reached the peak after around 24 h. In addition, it was found that most of the strains with a combination of the *mdh* gene and the *styAB* gene initiated by the constitutive promoters with different strengths had an increase in indigo production. The strain E215 harboring the vector pETP_T7_-styAB-*P_cat_*-*mdh* exhibited the highest indigo synthesis ability, and the indigo production of E215 was 787.25 mg/L after 24 h of fermentation, which was approximately 70-fold higher than that in wild strain *Pseudomonas putida* B3. Combined with the analysis of gene expression levels and enzyme activity, it was because the *mdh* gene was constructed to provide intracellular cofactor NADH and enhance the redox-cofactor rebalancing ability that the synthesis system of indigo was promoted after regulation.

## 4. Discussion

Previous works revealed that the *styAB* gene was the key enzyme of indigo biosynthesis, which was regulated by the intracellular reducing capacity. Therefore, we speculated that changing the expression of the *styAB* gene from inducible expression to constitutive expression and improving the redox-cofactor rebalancing ability would enhance the indigo production of recombinant strains. After different strategies of genetic manipulations, the *styAB* gene and *mdh* gene were successfully constitutively co-expressed by different promoters in *E. coli*, which obviously enhanced the monooxygenase activity and indigo production, as expected. With 2.0 g/L tryptophan added into fermentation medium as substrate for fermentation, the maximum yield of indigo in recombinant strains was up to 787.25 mg/L after 24 h of fermentation, which was nearly the highest indigo-producing ability using tryptophan as substrate in recent studies.

In this work, we used different strength and regulatory features of promoters for co-expression optimization of the *styAB* gene and the *mdh* gene by changing the inducible expression to constitutive expression, according to the characteristics of redox reaction in the synthesis pathway of indigo. The results indicated that regulating the expression level of the *mdh* gene and increasing the ratio of NADH/NAD^+^ could indeed improve the expression of the *styAB* gene and the indigo production, but only to a certain extent. Even though the expression level of the *mdh* gene was increased significantly and the ratio of NADH/NAD^+^ raised correspondingly in strains E214 and E216, which was higher than that in strain E215, the expression of the *styAB* gene and indigo production did not necessarily increase in strains E214 and E216. This may be due to the strong and exogenous gene promoter expression of the *mdh* gene, with the cofactors then ascending to enhance the other metabolic pathways, resulting in the reduction of *styAB* gene expression and indigo production. Furthermore, the redox reaction catalyzed by IMO was probably not the only limiting factor in the indigo biosynthesis pathway. Since indigo biosynthesis in *E. coli* is a cascade reaction, it was reasonable that the appropriate balance in the expression level of the *styAB* gene and redox-cofactor rebalancing ability was essential for enhancing the production of indigo. It revealed that gene expression optimization to enhance the indigo biosynthesis pathway is needed for comprehensive and systematic combinatorial methods, rather than simply replacing the promoter of other “strong” ones.

## 5. Conclusions

In this study, different strength and regulatory features of promoters were used for co-expression optimization of the *styAB* gene and the *mdh* gene, changing the inducible expression to constitutive expression. After combinatorial optimization of gene promoter for expression, indigo production was significantly enhanced in the recombinant strains. It fulfilled a feasible and economic pathway for the efficient production of indigo. The genetic manipulation strategy probably revealed a new insight into the construction of indigo biosynthesis cell factory for application.

## Figures and Tables

**Table 1:** Plasmids and strains used in this work.

Strains and Plasmids	Related Properties or Functions	Source
Strains		
*Pseudomonas putida B3*	Wild type, used to offer *styAB* gene	Laboratory collection
*E. coli*-BL21 (DE3)	Used as host strain	Tiangen Biotech, Beijing, China
E210	*E. coli*-BL21 (DE3) harboring pET-28a(+)	This work
E211	*E. coli*-BL21 (DE3) harboring pET-28a(+)-styAB	This work
E212	*E. coli*-BL21 (DE3) harboring pETP_T7_-styAB	This work
E213	*E. coli*-BL21 (DE3) harboring pETP_cat_-styAB	This work
E214	*E. coli*-BL21 (DE3) harboring pETP_T7_-styAB-*P_T7_*-*mdh*	This work
E215	*E. coli*-BL21 (DE3) harboring pETP_T7_-styAB-*P_cat_*-*mdh*	This work
E216	*E. coli*-BL21 (DE3) harboring pETP_cat_-styAB-*P_T7_*-*mdh*	This work
E217	*E. coli*-BL21 (DE3) harboring pETP_cat_-styAB-*P_cat_*-*mdh*	This work
Plasmids		
pET-28a(+)	Heterologous expression vector	MiaoLingPlasmid, Wuhan, China
pHS-avc	Used to offer *P_cat_* promoter	Laboratory collection
pET-28a(+)-styAB	pET-28a(+) carrying *styAB* gene originated from *Pseudomonas putida* B3, inducible expression	This work
pETP_T7_-styAB	pET-28a(+) carrying *styAB* gene ligated with *P_T7_* promoter, constitutive expression	This work
pETP_cat_-styAB	pET-28a(+) carrying *styAB* gene ligated with *P_cat_* promoter, constitutive expression	This work
pETP_T7_-styAB-P_T7_-*mdh*	pET-28a(+) carrying *styAB* gene ligated with *P_T7_* promoter and *mdh* gene ligated with *P_T7_* promoter, constitutive expression	This work
pETP_T7_-styAB-*P_cat_*-*mdh*	pET-28a(+) carrying *styAB* gene ligated with *P_T7_* promoter and *mdh* gene ligated with *P_cat_* promoter, constitutive expression	This work
pETP_cat_-styAB-*P_T7_*-*mdh*	pET-28a(+) carrying *styAB* gene ligated with *P_cat_* promoter and *mdh* gene ligated with *P_T7_* promoter, constitutive expression	This work
pETP_cat_-styAB-*P_cat_*-*mdh*	pET-28a(+) carrying *styAB* gene ligated with *P_cat_* promoter and *mdh* gene ligated with *P_cat_* promoter, constitutive expression	This work

## Data Availability

Data are contained within the article.

## Supplementary Materials

The following supporting information can be downloaded at: https://www.mdpi.com/article/10.3390/foods12030502/s1, Figure S1: The pathway of indigo biosynthesis from tryptophan in *E. coli*. Table S1: Primers used for PCR/RT-qPCR in this research.
