# Supplementary material for: A Combinational Optimization Method for Efficient Production of Indigo by the Recombinant Escherichia coli with Expression of Monooxygenase and Malate Dehydrogenase
Source: Foods. 2023 Jan 21;12(3):502. doi: 10.3390/foods12030502 (PMC9914922; doi:10.3390/foods12030502)
Supplement: Supplementary file 1 [file foods-12-00502-s001.zip › foods-2128023-supplementary.pdf]

Article

A Combinational Optimization Method for Efficient Production of Indigo by the Recombinant *Escherichia coli* with Expression of Monooxygenase and Malate Dehydrogenase

Zijing Pan, Dejiang Tao, Mingjing Ren and Lei Cheng *

Beijing Engineering and Technology Research Center of Food Additives, Beijing Advanced Innovation Center for Food Nutrition and Human Health, Beijing Technology and Business University (BTBU), Beijing 100048, China

* Correspondence: chenglei@btbu.edu.cn; Tel.: +86-10-68985252

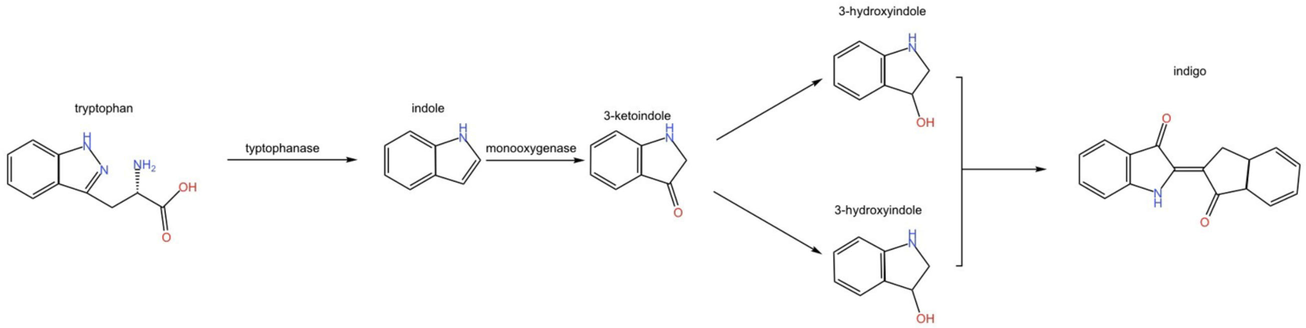

Figure S1. The pathway of indigo biosynthesis from tryptophan in *E. coli*.

Table S1. Primers used for PCR/RT-qPCR in this research

Primer	Sequence (5′-3′)
PCR	
F-styAB-BamHI	CGCGGATCCAGAGGATATCATCAATGAAAAAGCGTATCGGTATTG
R-styAB-HindIII	CCCAAGCTTTCAATTCAGTGGCAACGGGTT
F-P_{T7}-MluI	CGACGCGTCGATCCCGCGAAATTAAT
R-P_{T7}-XbaI	GCTCTAGAGGGGAATTGTTATCCGCTC
F-P_{cat}-MluI	CGACGCGTAAAAGTCCTCGATTCCTTCGATTT
R-P_{cat}-XbaI	GCTCTAGATGATCGGCACGTAAGAGGTTC
F-mdh-PCR	AGAGGATATCATCAATGAAAGTCGCAGTCCTCGGTTACTTATTAACGAACTCTTCGCCC
R-mdh-MluI-PCR	CGACGCGTCGTTACTTATTAACGAACTCTTCGCC
F-mdh-up-P_{T7}-MluI	CGACGCGTTTACTTATTAACGAAG
R-mdh-up-P_{T7}	CGGATAACAATTCCCCTCTCCTATAGTAGTTACTTTCAGCGTCAGGAG
F-mdh-down-P_{T7}	GTTCGTTAATAAAGTAACTACTATAGGAGAGGGGAATTGTTATCCGCTC
R-mdh-down-P_{T7}-SfoI	CCGCGGCGATCCCGCGAAATTAATAC
F-mdh-up–P_{cat}-MluI	CGACGCGTTCATTTATTAACGAAGAG
R- mdh-up- P_{cat}	CCTCGATTCCTTCGATTTTCTCCTATAGTAGTTCATTTATTAACGAACTCTTCGC
F- mdh-down- P_{cat}	GTTCGTTAATAAGTAAACCATTATAGGAGAAAATCGAAGGAATCGAGGAC
R-mdh-down- P_{cat}-SfoI	CCGCGGACTAGCCGTGCATTCTCCAAGG
F-pETP$_{T7}$-MluI-P_{T7}-mdh	AAGCAATTATTCATTTGCGCAATTATGCTGAGTGATATCCAGATCT
R-pETP$_{T7}$-SfoI-P_{T7}-mdh	ATCACTCAGCATAATCCGCGGGTTATGCGTTTGGCGGAGA
F-pETP$_{cat}$-MluI-P_{cat}-mdh	AAGCAATTATTCATTTGCGCAACTAGCCGTGCATTC
R-pETP$_{cat}$-SfoI-P_{cat}-mdh	GAATGCACGGCTAGTCCGCGGGTTATGCGTTTGGCGGAGA
F-pETP$_{T7}$-MluI-P_{cat}-mdh	AAGCAATTATTCATTTGCGCAATTATGCTGAGTGATATCCAGATCT
R-pETP$_{T7}$-SfoI-P_{cat}-mdh	GAATGCACGGCTAGTCCGCGGGTTATGCGTTTGGCGGAGA
F-pETP$_{cat}$-MluI-P_{T7}-mdh	AAGCAATTATTCATTTGCGCAACTAGCCGTGCATTC
R-pETP$_{cat}$-SfoI-P_{T7}-mdh	ATCACTCAGCATAATCCGCGGGTTATGCGTTTGGCGGAGA
qRT PCR	
F-styAB	GGCGAGCTGATTGAGATTC
R-styAB	TGCTGAAGAATGCCGATAA

F-mdh	CAGTTGCTGCGAAGAAAGG
R-mdh	TGTCGCTTGTGGTTTGTGAT
F-16s	CCACCTGGACTGATACT
R-16s	GCACCTGTCTCAATGTT
